# A Practical Treatise on the Diseases of the Sexual Organs of Women

**Published:** 1866-08

**Authors:** 


*A Practical Treatise on the Diseases of the Sexual Organs of Women.* By F. W. VON SCANZONI, Professor of Midwifery and Diseases of Females, in the University of Wurzburg, etc. (Translated from the French, and annotated by Augustus R. Gardner, M. D., New York.)

This is the title of a work lately received from the publisher, Robert M. DeWitt, New York.

We learn the work has been published for several years, but has not yet, to any extent, fallen into the hands of Southern readers, on account of the recent war. We do not know, from the publication itself, when it issued from the press, but infer, from the entry for copyright, in 1861, that about that year it made its appearance to the American reader in his own language. Throughout the work we find additions and comments, interspersed by the translator.

We feel warranted in stating, even from the imperfect perusal allowed, that no medical work, which it has been our privilege to examine recently, will afford more practical information to the physician than this. It is not our purpose to write an extended review of the book, nor shall we be satisfied with what may seem to our readers as a *formal puff.*

No class of diseases has been less understood and so unsuccessfully treated, as that to which the author confines himself in the work before us. In the early part of our professional career, no disease was so unsatisfactorily managed as uterine affections, under the then usual system of medication. Practitioners then, with volumes before them on this subject, were compelled to grope their way in the dark, so far as the true pathology and rational treatment of certain of these diseases were concerned. The terms applicable to symptoms—the result of various and, in some instances, opposite pathological conditions—were used as the names of uterine diseases. Under these circumstances only occasionally could success be reasonably expected. It is only necessary to mention the single symptom, leucorrhœa, arranged and described in the catalogue of uterine diseases by the writers of less than thirty years ago, to show the utter darkness that surrounded the practitioners only the fourth of a century since.

Enterprising minds at this period necessarily sought practical facts, and a more rational course of treatment than was afforded by the text books of that day. Von Scanzoni, as exhibited in the Treatise before us, labored to perfect a more rational system of treating diseases peculiar to females.

We cannot too highly recommend this work to the practitioner, as well as to the student of medicine. The simplicity of arrangement, and plainness of style adopted by the author, prove his determination to afford practical facts and useful suggestions, rather than make a display of useless learning and an array of unmeaning names. In treating of *some* of the diseases which have the symptom, leucorrhœa, the author thus describes one of the most common, under the head of "Inflammation of the mucous membrane of the uterus":

"Like every organ covered with a mucous membrane, the uterus is, upon its internal surface, exposed to those catarrhal and croupy inflammations which generally affect mucous membranes. The former are ordinarily observed in the non-pregnant state, while the latter are more often observed in the puerperal condition."

In giving the pathological anatomy of acute catarrh of the uterine mucous membrane, we have the following:

"The principal changes take place in the internal surface of the organ. The mucous membrane presents, principally in the portion which lines the uterine cavity, properly so called, an intense redness which is often spotted, the red places corresponding to the artificial openings of the utricular glands, which are surrounded by a fine capillary net-work, very strongly injected. Furthermore, the mucous membrane is very œdematous, softened, thickened, and projects in some points into the uterine cavity. It is easier than in the normal state to separate from the subjacent tissue larger or smaller shreds; and besides that, different portions of more or less extent are found deprived of their epithelium."

